# Using the MEAT VBP Framework to analyse and understand the value of surgical gloves: an explanatory case study

**DOI:** 10.1186/s13561-021-00325-z

**Published:** 2021-07-06

**Authors:** Benedict Stanberry, Gerhard Bothma, Katie Harrison

**Affiliations:** 1 IHLM, Oxford Centre for Innovation, New Road, Oxford, OX1 1BY UK; 2 Mölnlycke Health Care AB, Gamlestadsvägan 3C, 411 36 Gothenburg, Sweden

**Keywords:** MEAT VBP Framework, Value-based procurement, Surgical gloves, Case study

## Abstract

**Background:**

Value-based healthcare is being extensively piloted, scaled and implemented by healthcare providers and systems around the world. However, the ability of the healthcare supply chain function to strategically contribute to the improvement of value has been held back by a lack of practical tools for turning value-based procurement from concept into action. Two recently developed conceptual models – the American CQO Movement and the European MEAT VBP Framework – have been developed to support the implementation of value-based procurement in healthcare. We demonstrate how the latter of these models can be adapted and applied pragmatically to generate insights into the value of a specific medical device, technology or consumable.

**Methods:**

We undertook an explanatory, qualitative, single-case study focused on a specific consumable – surgical gloves – that provide a critical example of a type of medical device usually procured in high volumes but at risk of commoditisation due to a widespread lack of understanding of their value. Since the global Covid-19 pandemic prevented fieldwork, structured interviews were conducted via Zoom and corroborated by a literature review.

**Results:**

We identified ten cost criteria and eight outcome criteria with which the value of surgical gloves can be analysed and understood. For each of these criteria we propose definitions and value impact metrics that decision-makers can use during a procurement exercise to describe, quantify and compare glove value.

**Conclusion:**

The MEAT VBP Framework provides a highly practical and adaptable means of imposing both structure and rigour on a value analysis process and of qualitatively describing the potential value impact of surgical gloves for patients, professionals, providers and health systems.

## Introduction

At a time when most healthcare systems face unprecedented demand yet lack the resources to meet it the concept of value is steadily gaining increased prominence. First suggested by Michael Porter and Elizabeth Teisberg in 2006, value-based healthcare is a paradigm that enables healthcare providers to achieve a more optimal balance between the resources used to deliver care and the outcomes that matter to patients. According to Porter and Teisberg’s definition, the value of care is maximised when the best possible outcomes are delivered at the lowest reasonable cost over the full cycle of patient care [1].

There are significant similarities between the emergent concept of value-based healthcare and its older, more well-established sibling, cost-effectiveness analysis, which assesses value by comparing the costs and health benefits of two or more alternative treatments [2]. But although both value-based healthcare and cost-effectiveness analysis can be used to quantify the “bang for the buck” associated with different healthcare interventions, their respective definitions of value are different. In cost-effectiveness analysis value is an economic term assessed from a societal perspective by a specific decision-maker who must reach a view as to the desirability of certain health outcomes given the long-term costs that will be incurred in achieving them [3]. That value-based healthcare has achieved such rapid popularity shows, perhaps, that its patient-centred perspective and more short-term focus on specific episodes of care makes it more relevant to modern audiences than cost-effectiveness analysis and a more suitable lens through which to address contemporary challenges in the allocation of healthcare resources.

Since 2006, progressive healthcare providers have piloted, scaled and implemented value-based healthcare in at least three operational domains. Firstly, they have reconfigured their resources, including their people and facilities, in order to better match their capacity to demand and optimise utilisation [4]. Secondly, they have invested more wisely in the systems and technologies through which demand can be met without excessive capital expenditure [5]. Thirdly, they have tracked patient outcomes, measured the costs of the resources used to achieve those outcomes and used careful analyses of the resulting data to identify opportunities to enhance value [6]. However, despite the highly strategic role it plays in most hospital’s operations, it is only comparatively recently that the supply chain function has gained a more prominent and authoritative voice within the discourse on value-based healthcare [7].

### Value-based procurement models

For many decades the prevailing orthodoxy within most healthcare providers and systems has been that value is maximised primarily through price-based aggregation [8]. Hence a commonplace strategy for achieving economies of scale in product prices is the transfer of some or all procurement activities (eg, bidding, supplier evaluation, negotiation and contract management) to an independent entity that bulk buys on behalf of multiple providers [9]. Examples of such entities – known as Group Purchasing Organisations – include NHS Supply Chain in England [10], Réseau des Acheteurs Hospitaliers in France [11] and Premier, Inc. in the USA [12].

Today, almost all healthcare providers and systems understand what value and value-based healthcare are at a conceptual level – though there is significant variation between their capacities to actually implement it in day-to-day practice by identifying all the resources used to deliver care, calculating their costs and measuring the outcomes that matter to patients [13]. There is also a growing interest in the concept of value-based procurement but, until recently, little practical guidance was available to enable healthcare supply chain professionals to turn the concept into effective action at the individual, team or organisational level [14]. However, separate communities of practice with slightly different motivations but very similar aims have developed on opposite sides of the Atlantic to champion value-based procurement and provide practical assistance and support to supply chain professionals who want to implement it within their own health system or service.

### The Cost, Quality and Outcomes (CQO) Movement

**Fig. 1:**
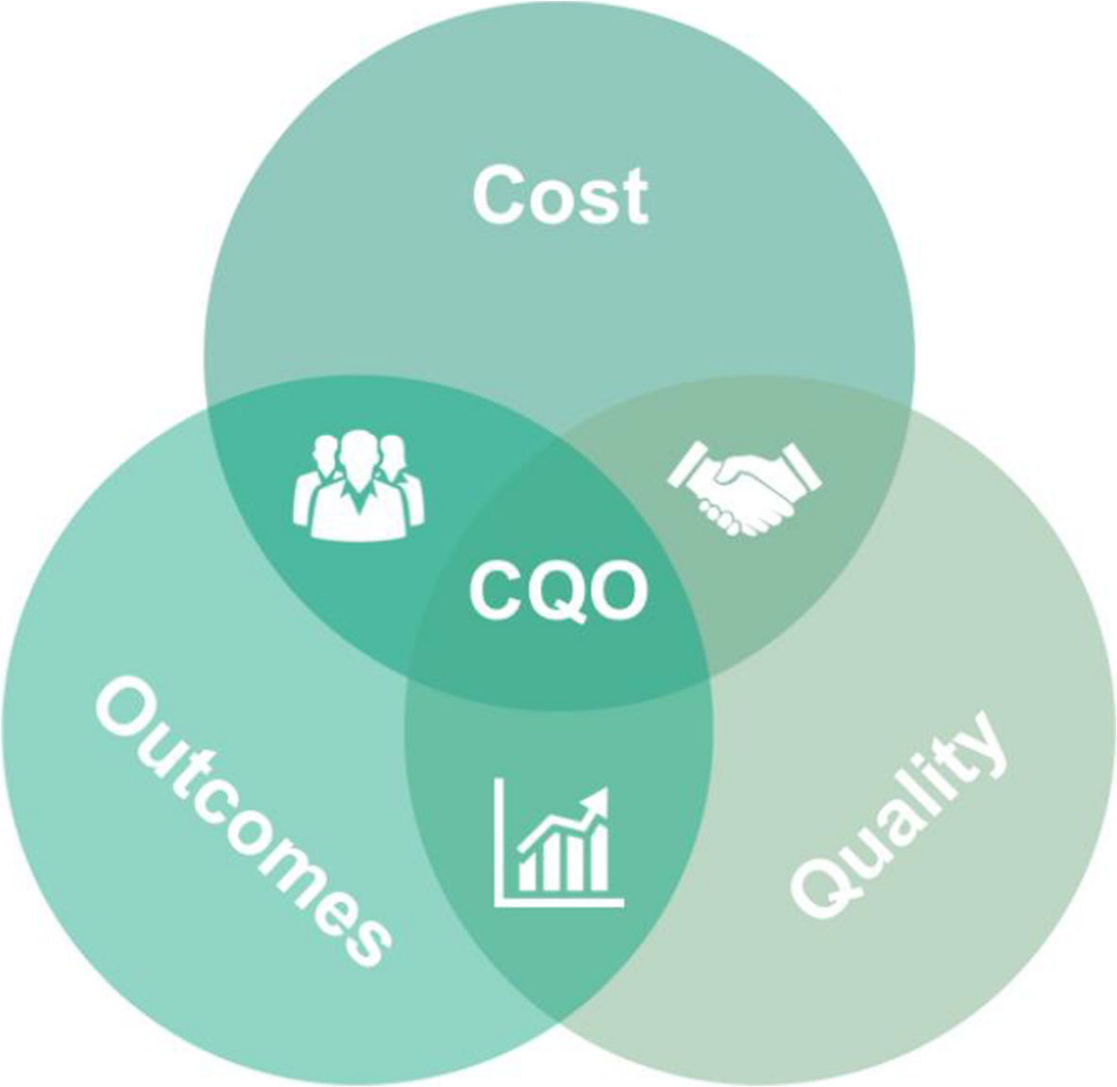
The Cost, Quality and Outcomes (CQO) Movement’s conceptual model

Recognising that the function of the healthcare supply chain needed to evolve alongside changes to US reimbursement policies the Cost, Quality and Outcomes (CQO) Movement was launched by the Association for Healthcare Resource and Materials Management (part of the American Hospital Association) in January 2013. Their conceptual model, shown in Fig. 1 below, encourages healthcare providers to take a more holistic view of the correlation between the costs associated with delivering patient care, the quality of that care and the resulting clinical and financial outcomes. In practice, this means encouraging providers to evaluate the clinical effectiveness of a medical device and to understand that if there is evidence the device delivers better outcomes than a lower-priced alternative, then using the more expensive device can potentially both enhance the value of care *and* deliver a greater financial return [15].

Among the progressive practices promoted by the CQO movement is the establishment of value analysis as the primary process through which purchasing decisions are made. In most American hospitals and health systems a physician-led, multidisciplinary value analysis committee or team is responsible for ensuring that the impact of a medical device on clinical outcomes, productivity, and patient and staff safety are carefully considered alongside its total cost. The higher the potential impact of a device on patient care, or a provider’s financial health, the more important it is that the device is properly assessed alongside potential alternatives [16].

The gradual adoption of value analysis has clear implications for the suppliers of medical devices to the US market. The multidisciplinary nature of value analysis committees and teams requires a more integrative value proposition that engages a broader range of stakeholders than just key opinion leaders. Moreover, suppliers are increasingly being expected to use succinct, structured presentations such as Value Analysis Briefs (VABs) to communicate the results of a systematic review of the likely clinical and economic benefits of a device and to provide evidence of its cost-effectiveness [17].

### The MEAT Value-Based Procurement (VBP) Framework

In 2014, the European Parliament adopted a new Directive that made significant changes to the rules around public procurement requiring that, henceforth, most contracts could only be awarded by publicly funded bodies on the basis of the “Most Economically Advantageous Tender” (MEAT). This meant that Europe’s public healthcare systems were now required to not only consider price during procurement exercises, but to also assess other factors such as cost-effectiveness, innovation, and environmental and social impact. The new Directive also acknowledged the need for more flexible tendering procedures by improving processes for competitive dialogue and negotiation [18].

**Fig. 2:**
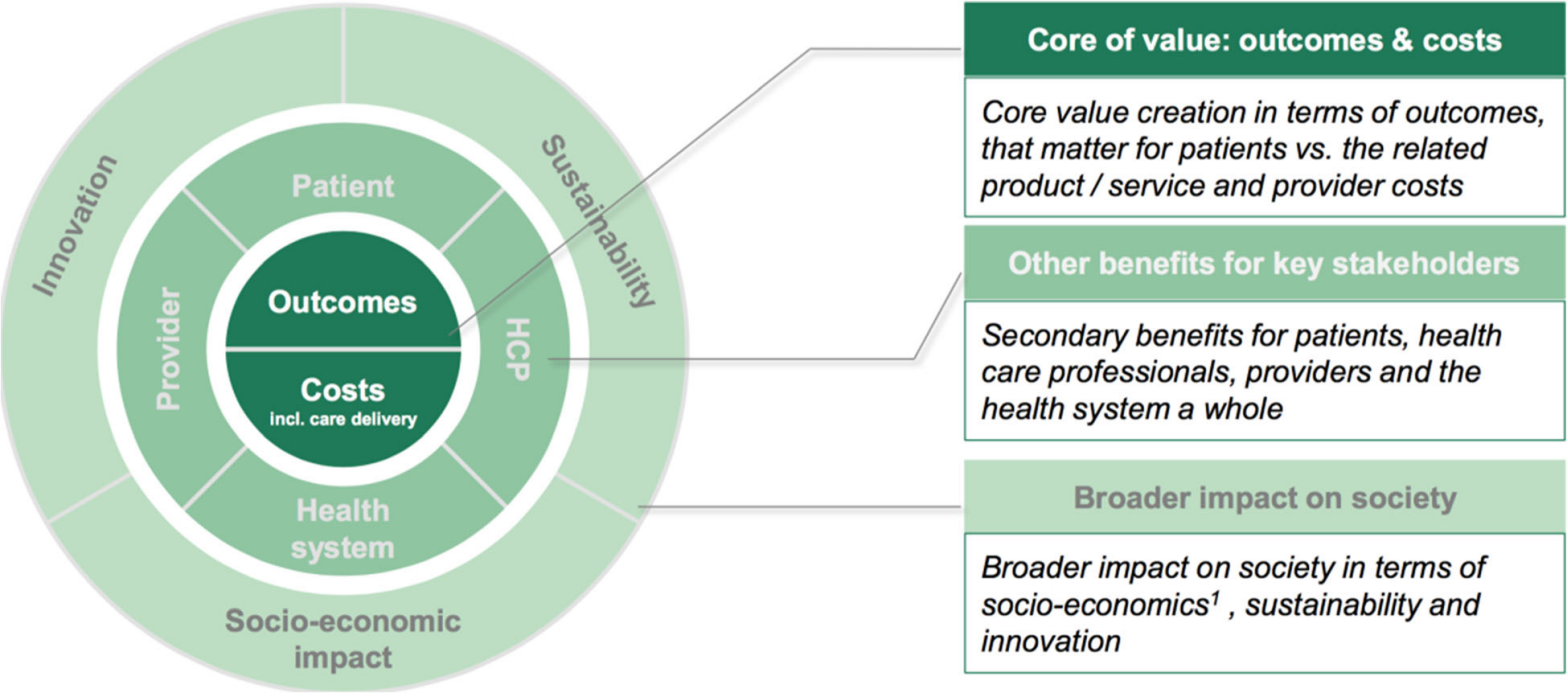
The MEAT Value-Based Procurement (VBP) Framework

MedTech Europe (a trade association) and Boston Consulting Group (a consultancy) have led the establishment of a community of practice that is similar to the American CQO Movement and comprised of a number of European healthcare procurement organisations. One of their first achievements has been to develop a detailed conceptual model known as the MEAT Value-Based Procurement (VBP) Framework – shown in Fig. 2 below [19].

The MEAT VBP Framework places Porter and Teisberg’s definition of value at its core and suggests numerous non-prescriptive criteria that can be used to quantify both the total costs associated with the procurement and use of a medical device over a full cycle of patient care and the outcomes it could contribute to – shown in Table 1 below. The outcomes that matter to patients are placed within the core of value alongside the total costs while any additional benefits for patients, as well as all the benefits for other key stakeholders, are placed in a secondary layer. Consideration of the broader impact of a procurement decision on society – in terms of socio-economics, sustainability and innovation – is encouraged through inclusion of these outcomes in a tertiary layer [20]. The MEAT VBP Framework has been piloted by early adopters across Europe in projects that have applied it to the procurement of many different types of devices – including “smart” hospital beds, knee implants, infusion pumps and anti-coagulation treatments [21, 22].

**Table 1:** Layers, categories and criteria in the MEAT VBP Framework

Layer		Category	Criteria
**Costs**	***Product***	**Purchasing**	1. Price of purchasing / renting product / solution
			2. Delivery and installation
			3. Conversion: staff training for new product
			4. Compatibility: upgrades to systems / infrastructure
		**Maintenance**	5. Spare parts
			6. Technical staff time
			7. Service contract
		**Storage**	8. Storage room / infrastructure
			9. Replacement at end of shelf life
		**Disposal**	10. Disposal / decommissioning
	***Care delivery***	**Operating / healthcare delivery**	11. Medical staff time using device
			12. Ongoing staff training
			13. Cost of consumables
			14. Infrastructure usage
			15. Unplanned usage: failure rate
			16. Power / gas usage
			17. Reprocessing costs
**Outcomes**		**Outcomes and evidence**	18. Evidence of relevant outcomes improvement
			19. Existence of high quality outcomes data
		**Outcomes focus**	20. Support in measuring and reporting on outcomes
			21. Willingness to offer outcomes-dependent risk sharing
**Other benefits for key stakeholders**		**Patients’ secondary benefits**	22. Patient and/or relative comfort and convenience
			23. Patient flexibility and mobility
			24. Impact on treatment adherence
		**Healthcare professional benefits**	25. Secure usage for care providers
			26. Ease-of-use / handling and functionality
			27. Training and access to education
		**Healthcare provider benefits**	28. Maintainability, warranty and technical service support
			29. Support improving efficiency along patient pathway
			30. Alignment and support with reimbursement structure
			31. Support on administration, storage or logistics
			32. Strategic fit for provider and support of strategy
		**Healthcare system benefits**	33. Reduced long-term costs of treatment
			34. Reduction of rehospitalisation / number of treatments
**Broader impact on society**		**Innovation**	35. Development of new and substantially improved technology
			36. Contribution to development of healthcare
		**Sustainability**	37. Environmental impact
			38. Socially responsible product value chain
		**Socio-economic impact**	39. Impact on people not in the workforce
			40. Burden carried by non-professional care providers
			41. Impact on competition in medical technology sector

### The models compared

The American CQO model and the European MEAT VBP Framework have evolved independently of each other. Each has been developed by a separate community of practice with nearly identical aims: to make value the over-riding objective of healthcare professionals, providers and systems, and to ensure the healthcare supply chain function plays its part in the establishment of value-based healthcare. However, the two models go about this in different ways and offer their users different benefits.

The CQO model offers a high-level conceptual representation of the trinity of aims – ie, cost, quality and outcomes – that progressive clinicians, supply chain professionals, executives and suppliers must optimise if they are to deliver better value healthcare. The model is unclear, however, regarding how these three variables should be defined, measured and optimised. Furthermore, its implementation in any given hospital seems to be highly dependent on the maturity and sophistication of that hospital’s value analysis function. Although detailed guidance on best practice in value analysis has become available in recent years [23, 24], the CQO model neither enables the potential value of devices to be quantified nor facilitates value comparisons between alternative devices so cannot drive greater consistency in the way value analysis is practiced by different providers and systems.

The European MEAT VBP Framework, on the other hand, provides a detailed yet common-sense framework for understanding and evaluating the value delivered by a specific medical device. It uses the same two layers of analysis – costs and outcomes – as those originally proposed by Porter and Teisberg, and has multiple descriptive categories and associated criteria within each layer. Although the Framework aims to facilitate greater consistency in the analysis of value, it still allows users the flexibility to use only those categories and criteria that are appropriate to the device being analysed – ie, those that accurately describe all the costs arising from its procurement and use, and all the outcomes it is expected to deliver for patients and other stakeholders. Unfortunately, the adoption of this Framework by Europe’s predominantly public healthcare systems has been limited – in part due to a lack of the structures and processes, such as value analysis, that have made it possible to implement value-based procurement practices in American hospitals.

This paper therefore uses a case study to demonstrate how the MEAT VBP Framework can be used to analyse and understand the value that a medical device or consumable can provide to patients, professionals, providers and health systems. We aim to demonstrate that using a detailed conceptual model such as the MEAT VBP Framework can make it relatively straight-forward for decision-makers to take a value-based approach to healthcare procurement and can potentially lead to different procurement choices than a purely price-based approach. In doing so we want to pragmatically contribute to current discussions on how the implementation of a value-based approach to procurement, with its attendant focus on both costs and outcomes, can enable the healthcare supply chain to play a much larger and more influential role in the implementation of value-based care.

## Methods

### Research design

Case studies are typically classified as either exploratory, descriptive or explanatory and have been described as the preferred method for research where “how” and “why” questions are being asked about contemporary phenomenon that are being studied within a real-life context over which the researcher has little or no control [25]. For this research we therefore chose to undertake an explanatory single-case study focused on a specific device – surgical gloves – because they are an extreme example of a type of medical consumable that, because they are so critical to the creation and maintenance of an aseptic barrier between patient and professional, are usually procured at high volume but are at risk of commoditisation – ie, being procured solely on the basis of price due to a widespread lack of understanding of their value.

### Surgical gloves

First used in 1889, surgical gloves made of rubber and covering the entire hand and wrist were originally only intended to protect surgeons and their staff from developing dermatitis from contact with the mercuric chloride that was used as a surgical disinfectant. It was not until 1894 that the pioneer of antiseptic surgery, Joseph Lister, began to routinely sterilise gloves. However, when this practice resulted in radical reductions in post-operative infections and the germ theory of disease became better and more widely understood, the use of gloves in aseptic surgery became commonplace [26].

Disposable, single-use gloves made of natural rubber latex have been widely available since the early 1960s and are now both clinically and economically preferable to reusable gloves, even in resource-constrained settings [27] – though a surge in the incidence of latex allergies among both patients and health professionals has led to the widespread use of synthetic alternatives such as polyisoprene [28]. “Double gloving” – ie, wearing two pairs of gloves – is becoming increasingly common since a Cochrane review indicated that this practice can significantly reduce the risk of infection due to glove failure or puncture [29].

### Data collection

The case study is not a method but a research design so provides scope for a multi-method design that combines both quantitative and qualitative data [30]. However, although the MEAT VBP Framework is designed to be used in both the quantitative and qualitative analysis of value we limited our data collection to qualitative sources because we lacked access to a healthcare provider or system from where we could collect the necessary cost data. We did, however, ensure that our data collection exercise identified all of the value impact metrics necessary to enable future researchers or practitioners who have access to detailed resource utilisation and unit cost data to use our research as the basis for a quantitative estimation of the value of surgical gloves.

Research involving on-site fieldwork in hospitals was not possible due to the global Covid-19 pandemic. We therefore used the 41 criteria in the MEAT VBP Framework as the basis for structured interviews conducted via Zoom with a global marketing manager with responsibility for and deep knowledge of both surgical gloves and the surgical gloves marketplace. These interviews were used to identify which cost criteria in the MEAT VBP Framework were implicated in the purchasing, maintenance, storage and disposal of surgical gloves and in their use for the delivery of care, as well as which outcome criteria could be used to describe their benefits for patients, professionals, providers and health systems.

Following these interviews a new version of the MEAT VBP Framework, as applied to surgical gloves, was generated and the validity of the layers, categories and criteria in our applied framework were tested through interviews with additional surgical glove marketing practitioners and procurement experts. The views expressed by the interviewees were further validated by a literature review of the evidence-based research on surgical gloves.

## Results

By carefully reviewing all 17 of the criteria contained in the cost layer of the MEAT VBP Framework we were able to identify and define ten criteria that apply to surgical gloves and seven criteria that do not. The applicable cost criteria described in Table 2 include purchase price, delivery or collection, staff training, infrastructure, storage, replacement of expired items, disposal, medical staff time, failure rate and infrastructure usage. For each of these cost criteria we have suggested value impact metrics that can be used to calculate value when the necessary cost data are available.

**Table 2:** Layers, categories and criteria in the MEAT VBP Framework applied to surgical gloves

Layer		Category	Criteria	Definition	Value impact metrics
**Costs**	***Product***	**Purchasing**	1. Price of purchasing / renting product / solution	The net price, inclusive of any discounts or rebates, paid for the volume of gloves purchased.	Number of units purchased (n) x Price per unit ($) x Discount (%).
			2. Delivery and installation	Any additional costs for delivery or collection of gloves and installing them in place.	Any extra costs charged for delivery. Any expenses incurred during collection and/or installation.
			3. Conversion: staff training for new product	Training and support costs required to convert to a new type of glove.	Any direct vendor training costs. Indirect costs: Staff wages ($/h) x Training duration (h).
			4. Compatibility: upgrades to systems / infrastructure	Costs of any changes to infrastructure or systems.	All costs arising from changes to infrastructure or systems.
		**Storage**	5. Storage room / infrastructure	All costs arising from storing surgical gloves.	Any fee for vendor-managed inventory ($/year). Storage capacity required (m^3^) x Cost of storage capacity ($/m^3^/year).
			6. Replacement at end of shelf life	Costs arising from replacing unused gloves at the end of their shelf life.	Number of units unused at end of shelf life (n) x Purchase price ($).
		**Disposal**	7. Disposal / de- commissioning	Costs of disposing of surgical gloves.	Weight of surgical gloves used (kg/year) x Disposal cost ($/kg). Any extra costs charged for collection, if applicable.
	***Care delivery***	**Operating / healthcare delivery**	8. Medical staff time using device	Any change gloves cause in the amount of time staff spend delivering healthcare.	Estimated additional or saved time per staff member per procedure (h) x Wages of each staff member ($/h) x Number of procedures (n).
			9. Infrastructure usage	Any change gloves cause in the utilisation of fixed assets – eg, operating rooms.	Estimated number of operating room ‘tear downs’ avoided (n) x [Average cost of a contaminated equipment disposed of ($) + Cost of sterilising reusable items ($) + OR overhead costs while idle ($)]
			10. Unplanned usage: failure rate	Cost of additional gloves used.	Estimated number of units failing (units / year) x Cost per unit ($).
**Outcomes**		**Patient benefits**	1. Reduction of risk of Surgical Site Infection (SSI)	Estimated reduction of the costs, both direct and indirect, of treating Surgical Site Infections (SSIs).	Estimated number of SSIs avoided (n) x Average additional cost incurred to treat a SSI ($).
			2. Reduction of risk of allergic or anaphylactic reactions	Estimated reduction of costs, both direct and indirect, of treating allergic or anaphylactic reactions.	Estimated number of allergic or anaphylactic reactions avoided (n) x Average additional cost incurred to treat a reaction ($).
**Other benefits for key stakeholders**		**Healthcare professional benefits**	3. Reduction of risk of occupational exposure to blood-borne pathogens	Estimated reduction of costs, direct and indirect, arising from occupational exposure to blood-borne pathogens.	Estimated number of occupational exposures avoided (n) x [Average additional cost incurred to treat an exposure ($) + Average cost of associated sick pay ($) + Average cost of associated locum cover ($)].
			4. Reduction of risk of allergic or anaphylactic reactions	Estimated reduction of costs, direct and indirect, arising from occupational exposure to latex.	Estimated number of allergic or reactions avoided (n) x [Average cost incurred to treat a reaction ($) + Average cost of associated sick pay ($) + Average cost of associated locum cover ($)].
			5. Ease-of-use / handling and functionality	Impact of gloves on surgeon dexterity and tactile sensitivity.	Surgical team satisfaction. Impact on productivity and/or performance. Standardised tests.
		**Healthcare provider and healthcare system benefits**	6. Training and access to education	Availability and provision of CPD / CME activities to surgical staff.	Training hours required (h). Contract compliance rate.
			7. Strategic fit for provider and support of strategy	Estimated reduction of costs or increase in revenue achieved by strategic projects in which switching to safer surgical gloves plays a significant role.	Estimated costs saved ($) + estimated additional revenues ($).
			8. Reduction of medico-legal claims	Estimated reduction of costs arising from defending and/or settling medical-legal claims.	Estimated number of medico-legal claims avoided (n) x Average amount of settlement or damages and associated legal costs ($).

We found many of the outcome and benefits criteria defined by the MEAT VBP Framework to be too broad and unspecific to be of practical help in describing the benefits of a specific type of medical device or consumable. However, using these criteria as a starting point we were able to identify and define eight specific outcome criteria to describe the benefits of surgical gloves. Five of these criteria described what we viewed as the fundamental benefits of surgical gloves that accrue to patient and healthcare professionals. A further three criteria related to attractive additional benefits that can accrue to healthcare providers and systems that procure high-value gloves. Value impact metrics have also been suggested for these outcome criteria.

### Purchasing costs

The first cost category in the MEAT VBP Framework covers all of the one-off costs relating to the procurement of surgical gloves. Naturally, these costs start with the net price, inclusive of any discounts or rebates, paid by the procurer for the volume of gloves purchased (cost criteria 1). If the glove supplier levies an extra charge for delivery, or requires the procurer to arrange collection, then this must be added to the purchase cost (cost criteria 2).

When a surgical team switches from one type of glove or gloving system to another some costs may be associated with the working time spent receiving training and support on the new glove from its vendor (cost criteria 3).

One frequently overlooked cost that might be incurred during purchasing can arise from any changes that are required to assure the compatibility of a new product or service with existing systems or infrastructure (cost criteria 4). Increasingly, however, hospitals are taking the strategic decision to become latex free environments and are switching from natural rubber latex to synthetic gloves. This usually reduces infrastructure costs by not only removing the need for pre-operative screening for latex allergies but also eliminating the need to insure against the risk of such allergies [31].

### Maintenance costs

This second category in the cost layer considers all the costs that have to be incurred to keep a product or service in working order during its operational lifetime, however long that may be. This may include the costs of the time a hospital’s technical staff spend maintaining a product or, if this function is outsourced, the costs of a service contract. There could also be continuing costs associated with buying spare and replacement parts. However, none of these costs arise in relation to medical consumables such as surgical gloves.

### Storage costs

This third category in the cost layer considers all the expenses arising from storing surgical gloves (cost criteria 5) – whether onsite or off, outsourced or insourced – including the cost of any gloves that remain unused at the end of their shelf life (cost criteria 6).

### Disposal costs

The fourth cost category in the Framework considers the one-time costs arising from the disposal or decommissioning of a product. Since all used surgical gloves are clinical waste they must be correctly bagged, marked and secured before being sent for incineration. Most waste management services charge healthcare providers by the kilogramme to collect and dispose of their clinical waste legally and safely (cost criteria 7).

### Operating / healthcare delivery costs

Within the broad category of costs that arise from actually delivering care using a medical device, there are several criteria suggested in the MEAT VBP Framework that do not apply to our analysis of surgical gloves. For instance, disposable gloves do not require the purchase of any further consumables in order to use them safely. They do not incur sterilisation or reprocessing costs, nor do they require electricity or gas. Moreover, if a surgical team has been properly supported during their transition to a new type of glove or gloving system then further training is unlikely to be needed. Our analysis has, however, identified three important cost criteria that fall within the healthcare delivery category as it applies to surgical gloves.

Firstly, almost all medical products have the potential to either improve or erode the productivity of healthcare staff. Surgical gloves that slow a task or process down or whose poor reliability leads to tasks being repeated or duplicated because of failure are net destroyers of value because they increase staff labour time, every minute of which carries an employment cost. Conversely, gloves that speed up a task or process, or which fail less often, release valuable time back to medical staff so are net contributors of value. Hence surgical gloves may, over time, increase surgical team productivity (cost criteria 8).

Surgical gloves don’t only influence staff productivity – they can also have a tangible impact on the productivity of fixed assets – particularly a provider’s most in-demand resources such as operating rooms (cost criteria 9). Since the overhead costs of such assets are largely fixed their efficiency is dependent upon utilisation – ie, the number of surgical procedures they can accommodate during a defined period of time. Latex proteins can contaminate all items that are touched in the operating room, requiring a complete new set-up before a patient with (or suspected of having) a latex allergy is operated on. Many hospitals are therefore making strategic decisions to become latex free in order to improve the productivity of their most expensive infrastructure, their operating rooms, by reducing the waste associated with the disposal of single use items and the sterilisation of reusable items that have become latex contaminated.

The final cost driver we have identified is one that comes into play every time a surgical glove fails and has to be replaced in order to maintain planned inventory levels (cost criteria 10). If a glove perforation is detected and remedied without harm occurring to either the patient or the wearer then the doffing and disposing of the failed glove, rescrubbing if necessary, and donning of a new glove will have an impact on medical staff time (cost criteria 8) and require the unplanned consumption of additional gloves (cost criteria 10). As we will discuss, however, the risk of Surgical Site Infections (SSIs) and possible exposure of healthcare workers to blood-borne pathogens means that the consequences of unplanned failure of a surgical glove go far beyond just this category of costs.

### Outcomes and benefits for patients

Surgical gloves are essential to aseptic surgery and while occasional glove failures may have negligible consequences for inventories they can have very significant consequences for patient safety. Clinically visible perforations (caused by needlestick punctures or sharp surfaces) and pinholes (a defect that can affect all types of surgical gloves) occur frequently [32]. Their risk increases with the duration of surgery [33] and they are associated with an increased likelihood of pathogens being transferred from a surgical team-member to a patient and vice-versa [34].

SSIs are now the most common hospital-acquired infections among surgical patients and surgical glove perforation can increase the risk of SSIs [33]. A recent systematic review of the impact of SSIs on European hospitals has estimated that the total medical costs for a patient with a SSI can be up to €30,000 higher than for a non-infected patient. These costs arise from the patient’s extended length of stay, as well as from additional laboratory testing, medication and readmission [35]. Strategies that reduce the risks of surgical glove failure can therefore benefit both the clinical safety of patients and reduce costs by lowering the incidence of SSIs (outcome criteria 1).

Common strategies to reduce the risk of surgical glove failure and SSIs include double-gloving [36], changing gloves approximately every 90 minutes [37] and procuring high quality gloves from the outset. An international quality standard – the Acceptable Quality Level (AQL) – helps hospitals compare the cost and quality of surgical gloves by describing the maximum number of defective gloves that are permitted in a batch. Under the EN455 standards surgical gloves must have an AQL of 0.65 and examination gloves require an AQL of 4.0. The lower the AQL, the lower the manufacturer’s tolerance for defects such as pinholes that are identified during the random sampling of glove batches prior to their release.

Patients also benefit from measures that procurers take to reduce the likelihood of allergic or anaphylactic reactions to the proteins retained in natural rubber latex (outcome criteria 2). Patients at high risk of having a latex allergy include those who have had multiple previous surgeries, who have spina bifida, who have a history of environmental or food allergies, or of hand dermatitis, or who are employed in occupations where they are frequently exposed to latex [38]. The only way to manage such patients is to avoid the use of latex gloves in favour of synthetic alternatives or to ban latex gloves entirely [39].

### Outcomes and benefits for healthcare professionals

It is not only patients who are exposed to potential harm by surgical glove perforations and pinholes. Occupational exposure of healthcare workers to blood-borne pathogens such as HIV and Hepatitis B and C are a growing concern. In one survey, 95% of the surgeons polled said they had experienced at least one needlestick injury during their career [40]. Moreover, a recent systematic review of the costs of needlestick and sharps injuries among healthcare personnel found that such injuries could cost up to $1,691 per incidence. This includes the direct costs to providers and health systems of laboratory testing and treatment, the time and wages diverted to receiving or providing exposure-related care, lost productivity, staff absence and the payment of compensation [41]. Double-gloving is proven to reduce the incidence of needlestick injuries and can therefore improve the occupational health and safety of surgical teams (outcome criteria 3) [36].

Latex allergies also affect healthcare professionals. One study has estimated that, on average, approximately 17% of health professionals and 38% of dental personnel are allergic to latex [42]. So in addition to the physical discomfort and inconvenience professionals may experience due to latex allergies there may also be economic impairment caused by periods of sickness absence or being unable to continue working, resulting in a loss or reduction of their income. Switching to all-synthetic environments may therefore offer significant benefits for the occupational health of surgical teams (outcome criteria 4) [39].

Though the primary use of surgical gloves is to prevent the transmission of pathogens between patients and healthcare professionals, their impact on manual performance is also important since this affects surgical safety and efficiency. Gloves that provide minimum diminishment of dexterity and tactile sensitivity offer a measurable benefit to the surgeon and other members of the surgical team. This benefit can be evaluated using standardised tests (outcome criteria 5) [43].

### Outcomes and benefits for healthcare providers and systems

The potential availability of clinical advisers in an operating theatre, and the quality of the training and support they can provide, may have a significant impact on the willingness and ability of a surgical team to transition to a new type of surgical glove and the ability of a provider to comply with a supplier contract (outcome criteria 6).

Surgical gloves can play a vital role in enabling hospitals and healthcare systems to achieve strategic goals (outcome criteria 7). Switching to safer surgical gloves can facilitate progress towards implementing the International Patient Safety Goals that help accredited facilities address specific areas of concern in some of the most problematic areas of patient safety – including safer surgery and reducing the risks of healthcare-associated infections. A move to synthetic gloves may also, as we have previously discussed, enable providers to improve operating room productivity and enhance the occupational health and safety of surgical staff.

Both providers and systems may also derive significant medico-legal benefits by reducing the likelihood of having to settle patient claims for clinical negligence arising from SSIs and latex allergies or employee claims for failure to provide a safe working environment following exposure to blood-borne pathogens or long-term exposure to latex (outcome criteria 8).

## Discussion

Though its designers advise that the layers and categories of the MEAT VBP Framework be used consistently, they encourage its users to be flexible when selecting cost and outcome criteria by using only those criteria that are appropriate to the consumable, device or technology being analysed and the situation in which it will be used. So although the original Framework contains 17 cost criteria and 24 outcome criteria, our use of just ten relevant cost criteria and eight relevant outcome criteria to describe the value of surgical gloves was within the designer’s intent. However, applying the Framework to surgical gloves has revealed at least two redundancies.

**Fig. 3:**
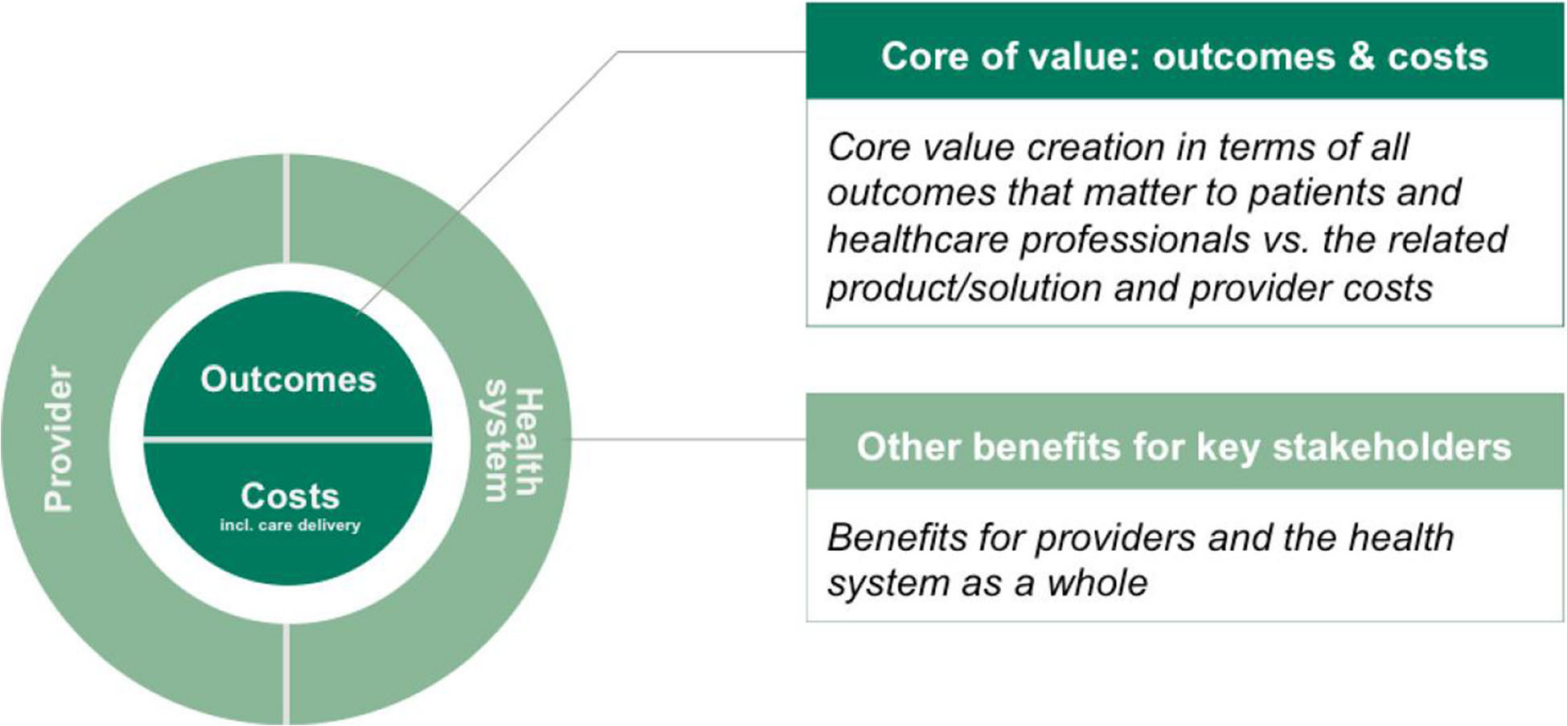
The MEAT VBP Framework applied to surgical gloves

Firstly, though we concede that the Framework’s patient-centredness may be enhanced by placing only the outcomes that matter for patients in the core of value, in surgical glove procurement the aseptic barrier is as important for healthcare professionals as it is for patients. We therefore feel that surgical gloves are one of many types of medical consumable for which the Framework should place the benefits for healthcare professionals in the core of value together with *all* of the benefits for patients. Secondly, because exploration of the economic or social consequences of glove procurement for society as a whole is beyond the scope of this research – as is any investigation of the effect of current procurement practices on glove innovation or the environment – the tertiary layer of the Framework was redundant in our analysis. Practitioners may therefore find a more simplified and “slimmed-down” version of the Framework – such as that suggested in Fig. 3, below – to be quicker and easier to apply.

The biggest frustration we encountered in undertaking this case study was our lack of access to the detailed cost data required to use the MEAT VBP Framework quantitatively. It is in the claimed ability of the Framework to actually calculate value that it makes both its boldest claim and faces its greatest challenge – so our lack of access to cost data was a significant limitation to this current study that must be addressed in future studies. In Table 2 we suggested value impact metrics as simple formulae for calculating specific cost or outcome criteria but did not have an opportunity to actually perform these calculations. We therefore suggest that further studies of the MEAT VBP Framework should use micro-costing (ie, the direct enumeration and costing of every input consumed in the treatment of a particular patient) to measure costs and outcomes as accurately as possible [44]. Accurate measurement of each resource identified in the value impact formulas in the right-hand column of Table 2 could be undertaken using appropriate data collection tools such as standardised comprehensive templates, targeted questionnaires and interviews, activity logs, direct observations (ie, time-and-motion studies) or on-site databases and records (such as cost accounting systems) [45]. Though these collection techniques can be time-consuming they would yield fairly precise cost data that could be fed into the value impact formulas to achieve an accurate valuation of each cost or outcome criteria that applies to the medical consumable under analysis. This methodology has already been shown to be of value in evaluating the cost of both specific surgical technologies and surgical interventions in general [46, 47].

In the example shown in Table 3 below, we have taken the final step of validating our adapted version of the MEAT VBP Framework shown in Fig. 3 by applying it to a specific surgical glove – the Biogel® PI Indicator System manufactured by Mölnlycke Health Care AB [51]. The results in Table 3 demonstrate that the adapted Framework can be readily used to qualitatively describe the potential value impact of a specific type of surgical glove for patients, professionals, providers and health systems.

**Table 3:** The MEAT VBP Framework applied to the Biogel® PI indicator system

Layer		Category	Criteria	Value impact
**Costs**	***Product***	**Purchasing**	1. Price of purchasing / renting product / solution	The Biogel® synthetic double-gloving system has a price that reflects its high quality and performance.
			2. Delivery and installation	Delivery and installation costs are typically included in the final purchase price by Biogel® distributors. For the past 4 years, over 99% of Biogel® inventory has been available at all times, reducing the impact of back orders.
			3. Conversion: staff training for new product	Biogel® clinical advisers are available to provide real time support in the Operating Room at no additional charge and will enable the surgical team to choose the glove material, size and features that are right for them.
			4. Compatibility: upgrades to systems / infrastructure	Synthetic Biogel® gloves will support the creation of a latex-free environment with cost-saving arising from elimination of patient allergy screening.
		**Storage**	5. Storage room / infrastructure	Biogel® undergloves and overgloves are supplied in a single pack to reduce storage space.
			6. Replacement at end of shelf life	Assistance is available to remove outgoing gloves and enable conversion to Biogel®.
		**Disposal**	7. Disposal / de- commissioning	Negligible impact on clinical waste management costs.
	***Care delivery***	**Operating / healthcare delivery**	8. Medical staff time using device	Biogel® double-gloving systems support staff productivity by being easier to don; less likely to fail during use; making punctures visible if they do occur; and not requiring the anatomical surgical scrub process to be repeated if only the outer glove fails.
			9. Infrastructure usage	All Biogel® gloves are air-inflated and visually inspected before packing, enabling defects occurring during the packing process to be detected and delivering the lowest in-use failure rate of any surgical glove manufacturer [48].
			10. Unplanned usage: failure rate	Made from polysoprene and free from any chemical accelerators, synthetic Biogel surgical gloves fully support the elimination of latex from the Operating Room, therefore reducing the time lost to tear-downs.
**Outcomes**		**Patient benefits**	1. Reduction of risk of Surgical Site Infection (SSI)	Biogel® gloves have an AQL process average of 0.20 versus an industry standard of 0.65 resulting in the lowest in-use failure rate of any surgical glove manufacturer [48]. A patented Puncture Indication System combines a coloured underglove with an opaque overglove, highlighting trapped fluid and making perforations and punctures highly detectable [49]. Both these features significantly reduce the risk of glove failure and associated SSIs.
			2. Reduction of risk of allergic or anaphylactic reactions	Biogel® synthetic gloves are free from natural rubber latex, reducing the risk of allergic or anaphylactic reactions or sensitivity due to frequent or prolonged exposure.
**Other benefits for key stakeholders**		**Healthcare professional benefits**	3. Reduction of risk of occupational exposure to blood-borne pathogens	Double-gloving using surgical gloves that provide a Puncture Indication System reduces the risk of sharps injuries and exposure to blood-borne pathogens by up to 71% [49].
			4. Reduction of risk of allergic or anaphylactic reactions	Biogel® synthetic gloves are free from natural rubber latex, reducing the risk of allergic or anaphylactic reactions or sensitivity due to frequent or prolonged exposure.
			5. Ease-of-use / handling and functionality	This type of glove is manufactured for a comfortable fit and precise feeling [50].
		**Healthcare provider and healthcare system benefits**	6. Training and access to education	Regular CPD/CME accredited clinical training is available for Biogel® users
			7. Strategic fit for provider and support of strategy	Biogel® gloves can play a significant role in strategic projects focused on implementing the International Patient Safety Goals, improving operating room productivity, or enhancing the occupational health and safety of healthcare workers.
			8. Reduction of medico-legal claims	Synthetic glove such as Biogel® which have a very low risk of perforation or pinhole defects support reduction of SSIs, patient allergies and occupational exposure to pathogens and latex. Reduced occupational exposure to needlestick injuries and early detection reduces the risk of litigation following the occurrence a clinical adverse event.

## Conclusion

The healthcare supply chain function has a vital role to play in the implementation of value-based healthcare by facilitating and promoting value-based procurement practices. Such practices cannot take root or grow in a procurement culture whose primary goal is price reduction. Fortunately, there are growing communities of practice, in both the USA and Europe, that are helping providers and health systems to build discipline and data into the way they evaluate and procure medical devices and consumables. The American CQO Movement and the European MEAT VBP Framework are the two leading conceptual models that take a holistic approach to value. The CQO Movement encourages investment in the structures and processes that support the implementation of value-based procurement at the hospital level while the MEAT VBP Framework enables value-based decision making by all who are involved in the healthcare supply chain. Used qualitatively, the MEAT VBP Framework is capable of delivering meaningful insights into the value of a medical consumable such as surgical gloves and enabling side-by-side comparisons of the value of different glove types. Used quantitatively with the necessary cost data, this framework will likely make it possible to use micro-costing techniques to synthesise clinical and financial evidence into a compelling business case demonstrating the value of any medical device or consumable.

## Data Availability

All data generated or analysed during this study are included in this published article.

## Abbreviations

CQO: Cost quality outcomes
MEAT: Most economically advantageous tender
VBP: Value-based procurement
NHS: National Health Service
USA: United States of America
VAB: Value analysis brief
AQL: Acceptable quality level
SSI: Surgical site infection
HIV: Human Immunodeficiency Virus
AB: Aktiebolag
